# County-Level Economic Changes and Drug Mortality in the United States: Evidence from the Great Recession

**DOI:** 10.3390/ijerph192316261

**Published:** 2022-12-05

**Authors:** Yeonwoo Kim, Manuel Cano, Sehun Oh, Michael Betz

**Affiliations:** 1 Department of Kinesiology, University of Texas at Arlington, Arlington, TX 76019, USA; 2 School of Social Work, Arizona State University, Phoenix, AZ 85008, USA; 3 College of Social Work, The Ohio State University, Columbus, OH 43210, USA; 4 Department of Human Sciences, The Ohio State University, Columbus, OH 43210, USA

**Keywords:** Great Recession, community socioeconomic status, drug mortality

## Abstract

We aimed at examining whether county-level economic changes were associated with changes in county-level drug mortality rates since the Great Recession and whether the association is equally distributed across major sociodemographic subgroups. Using the Centers for Disease Control and Prevention’s Wide-Ranging Online Data for Epidemiologic Research (2004–2019), combined with census data, we conducted fixed effects analyses by including county-level economic changes as primary exposures and county-level drug-related mortality rates (per 100,000 people) from 2004–2007 (i.e., prior to the recession) to 2008–2011, 2012–2015, and 2016–2019 as an outcome variable based on 1833 counties. Our findings showed that drug mortality rates increased from 13.9 (2004–2007) to 16.0 (2008–2011), 18.0 (2012–2015), and 23.0 (2016–2019). Counties experiencing smaller median household income growth during and/or after the recession were associated with greater increase in drug mortality than counties experiencing larger median household income growth among the total population and all sociodemographic subgroups. Counties experiencing larger increases in unemployment rates and percentage of vacant housing units were associated with greater increase in drug mortality than counties experiencing smaller or no increase in unemployment rates and percentage of vacant housing units among certain sociodemographic subgroups. Findings suggest the importance of local economic contexts in understanding drug mortality risk since the recession. Drug overdose prevention polices need to be formulated by taking local economic changes following a major recession into consideration.

## 1. Introduction

In the United States (US), drug-related mortality has tripled over the past two decades, making it a leading cause of death [1]. As of 2019, the number of drug-related deaths was nearly twice the number attributed to motor vehicle accidents and four times the number of deaths attributed to firearm-related homicides [1]. The US opioid epidemic was initially driven by drastic increases in prescription opioid overdose deaths in the early 2000s [2]. More recently, greater availability of heroin and synthetic opioids led to subsequent surges in opioid-involved deaths in the 2010s [3,4]. Since then, stimulant-related overdose deaths (including cocaine and methamphetamine) have been driving recent increases in drug-related mortality [5].

Previous work has demonstrated striking geographical disparities in drug-related mortality rates, ranging from 4.7 to 123.2 per 100,000 persons across US counties [1]. The varying burden of drug-related mortality is shaped by a complex and changing interplay between geographic differences in socioeconomic conditions, subnational policies, and drug markets [6]. Economic conditions at the county level have lately drawn attention as key social determinants of geographical disparities in drug mortality. County-level economic disadvantage could impact drug mortality disparities via multiple pathways including residents’ access to health-related resources and interventions [7,8]. In particular, disadvantages in labor markets (e.g., higher unemployment rates) and housing conditions (e.g., greater vacant homes) are considered key predictors of adverse health outcomes [9,10,11,12,13].

The Great Recession, which occurred between December 2007 and June 2009, was a long and extensive economic downturn in the US. The effects of the Great Recession on local economies lingered and were pervasive even after 2009, transforming economic contexts for both individuals and communities [14]. In particular, low-income communities were hit hardest, where residents were more likely to have unstable jobs and higher proportions of subprime loans were given prior to the recession [15,16]. The disparate impact of the recession on local economies perpetuated economic inequalities across counties [17]. For example, communities that had high unemployment rates before the recession experienced larger increases in unemployment rates compared to similar communities with lower pre-recession unemployment rates [17]. Futhermore, the median income decreased in communities at the bottom 10 percent of the metropolitan income distribution from 2000 to 2010, while the median income during the same period increased in the communities at the higher end of the income distribution [15]. At the same time, vacant housing has been disproportionately accumulated in lower-income communities following a historically high level of foreclosures from the recession [18,19].

Widening economic disparities across local economies may bring about county-level variations in drug mortality. As county governments develop and implement strategies to reduce drug misuse and harm in their communities [7,8], counties that experienced reductions in tax revenues during and after the recession had limited funding to develop and maintain social services for substance misuse prevention [20]. Furthermore, counties hardest hit by the recession are also more likely to have adverse social environments, such as low social cohesion, social control, social support, and safety issues [21,22], which are associated with the risk of drug misuse [23].

Regarding the effects of county-level economic conditions on drug misuse, evidence suggests that greater unemployment rates at the county level are associated with increased prescription opioid use disorder at the individual level, especially among working-aged White males with lower educational attainment [24]. As a similar measure of economic condition, median household income at the county level is also considered an important predictor of drug mortality. For instance, Nosrati and colleagues [12] found that one standard deviation reduction in median household income per county was associated with a 13% increase in drug mortality. Vacant housing has also been examined as a predictor of drug overdose-related outcomes [11,25]. Social disorganization theory and scant literature suggest that vacant houses can provide potential space for illicit drug trade and consumption, raising drug overdose risks in the communities [26,27].

It is unclear to what extent the Great Recession may have impacted US counties’ trajectories of drug overdose mortality rates, considering mixed findings from prior research on recession-related changes in health behaviors and health outcomes [28,29,30,31,32]. Recessions have been linked to reductions in all-cause mortality yet increases in mortality attributed to specific causes of death, including poisoning, with patterns shifting over time [33,34,35]. While opioid-related emergency department visits in New York City declined in the short-term following the Great Recession [36], unemployment increases during the Great Recession were associated with rising rates of drug overdose mortality among working-age adults in metropolitan US areas [37]. To our best knowledge, there are no studies examining the association between local economic changes since the Great Recession period and geographical disparities in drug-related mortality rates over a decade and across different population subgroups. This examination may give insights into the extent to which new economic contractions due to the COVID-19 pandemic along with the lingering effects of the Great Recession influence geographical disparities in drug-related mortality. In addition, as there is recent evidence for the surge in drug mortality, the literature needs to be extended by drawing on the latest data and examining the drug mortality trends in connection with local economic contexts over time.

Considering these gaps in the literature, we first examined the recent county-level drug-related mortality before, during, and following the Great Recession to capture potential heterogeneous relationships across time periods as the opioid crisis has evolved. Second, we examined the associations between county-level economic changes since the Great Recession and drug mortality changes from 2004–2007 through 2016–2019. Given the Great Recession’s considerable impacts on labor and housing markets, we focused on unemployment, median household income, and vacant housing as key county-level economic factors.

## 2. Materials and Methods

### 2.1. Data

County-level death records for 2004–2019 were extracted from the National Center for Health Statistics’ underlying cause of death data files via the Centers for Disease Control and Prevention (CDC)’s Wide-Ranging Online Data for Epidemiologic Research (WONDER). Data are based on death certificates for US residents who died in the 50 states and the District of Columbia. Death-related information is reported by medical certifiers, such as physicians, chief medical officers of the hospital or nursing home, coroners, and medical examiners [38]. We pooled data for 4-year periods (i.e., 2004–2007, 2008–2011, 2012–2015, and 2016–2019) to increase the number of analytic counties and estimate reliable mortality rates because mortality rates are suppressed for counties with few drug-related deaths. The number of counties with valid drug mortality information accounts for more than 95% of all drug-related deaths in the US. The WONDER data was then linked to county-level socioeconomic data from the 2000 and 2010 Decennial Census and the American Community Survey (ACS) 2008–2012, 2012–2016, and 2016–2020 using the county FIPS code. After excluding counties that were missing data on county-level economic indicators and covariates, our final analytic sample for the total population included 1833 counties (2004–2007), 1761 counties (2008–2011), 1639 counties (2012–2015), and 1431 counties (2016–2019).

### 2.2. Measures

Drug Mortality Rates. Our outcome variable is county-level crude drug mortality rates per 100,000 people during each 4-year period (i.e., 2004–2007, 2008–2011, 2012–2015, and 2016–2019). Drug mortality was defined based on the International Statistical Classification of Diseases, 10th revision (ICD-10) codes: X40–44, X60–64, X85, Y10–14, and all other drug-induced deaths. We first examined county-level crude drug mortality rates per 100,000 individuals for the total population. We also estimated separate models for drug mortality rates by key population subgroups: age (15–34/44–54/55+), sex (male/female), and race/ethnicity (non-Hispanic Black/non-Hispanic White/Hispanic) based on documented sociodemographic variations in the association between county-level predictors and drug mortality [9].

County-Level Economic Status. Our primary exposures are three time-varying county-level economic indicators: (1) the percentage of vacant housing units, (2) unemployment rates, and (3) median household income. The indicators were obtained from the 2000 Decennial Census and ACS 2008–2012, 2012–2016, and 2016–2020, and these were linked to drug mortality data files based on years of data collection. Specifically, we linked drug mortality data for 2004–2007 to the 2000 Decennial Census, drug mortality data for 2008–2011 to ACS 2008–2012, drug mortality data for 2012–2015 to ACS 2012–2016, and drug mortality data for 2016–2019 to ACS 2016–2020. We linked the 2000 Decennial Census, not the ACS 2004–2008, to mortality data for 2004–2007 because the ACS is available from 2006–2010, in which the ACS 2006–2010 is potentially at risk of reverse causality bias. Each economic indicator was standardized with a mean of zero and a standard deviation of one for easier interpretation in multivariate analyses.

Covariates. We included county-level demographic (i.e., percentages of non-Hispanic Whites, females, and people of ages 65 or older) and social factors (i.e., adults of ages 25 or older who had completed college) as time-varying covariates. Demographic factors were obtained from the 2000 and 2010 Decennial Census and ACS 2012–2016 and 2016–2020, and social factors were obtained from the 2000 Decennial Census and ACS 2008–2012, 2012–2016, and 2016–2020. All the covariates were standardized to have a mean of 0 and a standard deviation of 1 for easier interpretation in multivariate analyses.

### 2.3. Analysis

Statistical analysis was conducted in multiple steps using Stata 15.1. First, univariate analyses were conducted to examine county-level sociodemographic characteristics as well as unadjusted drug mortality rates before, during, and after the recession for the total population and sociodemographic subgroups. Second, we used fixed effects linear regression models to estimate the association between changes in county economic status and changes in drug mortality rates. We adjusted only for time-varying covariates because a fixed effects model removes all observed and unobserved time-invariant differences across counties [39]. We first included each measure of time-varying county economic status one at a time, time-varying county sociodemographic characteristics, and fixed effects for county and time periods corresponding to the death rates. Then, we added each interaction term between years and county economic status to the model.

## 3. Results

Descriptive statistics are presented in Table 1. Percentages of people ≥ 65 years, college graduates, vacant housing units, and median household income have increased from 2000 through 2016–2010. Unemployment rates were on the rise from 2000 through 2008–2012 and decreased to 5.4% in 2016–2020.

**Figure 1:**
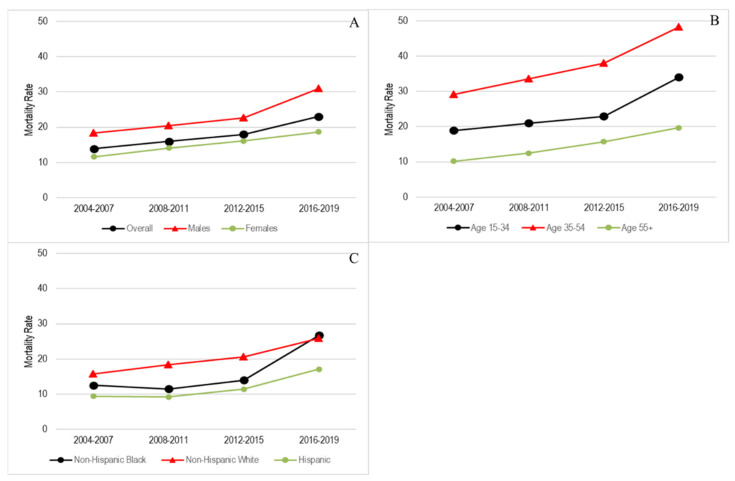
Unadjusted drug mortality rates among the total population and sociodemographic subgroups since the Great Recession, The Wide-Ranging Online Data for Epidemiologic Research, 2004–2019. Note: drug mortality rate indicates county-level crude drug mortality rates per 100,000 persons. (**A**) Drug mortality rates overall and by sex; (**B**) Drug mortality rates by age groups; (**C**) Drug mortality rates by race/ethnicity.

Figure 1 presents unadjusted average drug mortality rates in 2004–2007 (prior to the recession), 2008–2011 (during the recession), 2012–2015 (3–6 years after the recession), and 2016–2019 (7–10 years after the recession). Overall, as shown in Figure 1A, drug mortality rates per 100,000 people increased over time from 13.9 (95% CI: 13.5–14.3) to 16.0 (95% CI: 15.5–16.4) in 2008–2011, 18.0 (95% CI: 17.6–18.4) in 2012–2015, and 23.0 (95% CI: 22.4–23.5) in 2016–2019. The increasing trends were found in all sociodemographic subgroups with notable increases among males (see Figure 1A), individuals aged 15–34 years and 35–54 years (see Figure 1B), and non-Hispanic Blacks (see Figure 1C) from 2004–2007 to 2016–2019.

Table 2 presents the association between changes in county-level economic status and changes in drug mortality rates among the total population, males, and females. We observed that changes in unemployment rates and median household income were significantly associated with changes in drug mortality among the total population (*b* = 0.56, *p* < 0.05 and *b* = −2.25, *p* < 0.001, respectively), as shown in Models 2–3, Table 2. It indicates that when unemployment rates and median household income increased by one standard deviation (SD), drug mortality rates increased by 0.56 and decreased by 2.25, respectively. Changes in the percentage of vacant housing units were not significantly associated with drug mortality changes (*b* = −0.03, *p* > 0.05) (see Model 1, Table 2). In addition, changes in the percentage of vacant housing units and unemployment rates were significantly associated with drug mortality changes among females (*b* = 2.08, *p* < 0.001 and *b* = 0.68, *p* < 0.01, respectively) (see Models 1 and 2, Table 2), but not among males (*b* = 0.81, *p* > 0.05 and *b* = 0.61, *p* > 0.05, respectively). Changes in median household income were significantly associated with drug mortality changes among both males and females (*b* = −4.07, *p* < 0.001 and *b* = −2.08, *p* < 0.001, respectively) (see Model 3, Table 2), which indicates that when median household income increased by one SD, drug mortality rates per 100,000 people decreased by 4.07 for males and by 2.08 for females.

Table 3 presents the association between changes in county-level economic status and changes in drug mortality rates by three age groups. As shown in Model 1, changes in the percentage of vacant housing units were significantly associated with changes in drug mortality rates only among individuals aged 35–54 years (*b* = 6.14, *p* < 0.001). Changes in median household income were negatively associated with changes in drug mortality rates among all three age groups ([Age 15–34] *b* = −6.32, *p* < 0.001, [Age 35–54] *b* = −4.88, *p* < 0.001, [Age 55+] *b* = −1.34, *p* < 0.05) (see Model 3, Table 3). Unemployment rates were not significantly associated with drug mortality among all three age groups at alpha 0.05 (see Model 2, Table 3).

Table 4 presents the association between county-level economic changes and drug mortality changes by three racial/ethnic groups. Changes in the percentage of vacant housing units were significantly associated with drug mortality changes only among Hispanic individuals (*b* = 2.79, *p* < 0.05) (see Model 1, Table 4), and changes in unemployment rates were significantly associated with drug mortality changes only among non-Hispanic White individuals (*b* = 0.73, *p* < 0.01) (see Model 2, Table 4). As shown in Model 3, changes in median household income were negatively associated with drug mortality changes among all three racial/ethnic groups ([non-Hispanic Black] *b* = −4.74, *p* < 0.001, [non-Hispanic White] *b* = −2.91, *p* < 0.001, [Hispanic] *b* = −5.14, *p* < 0.05), which implies that an increase in median household income was associated with decreased drug mortality rates.

Collectively, changes in the percentage of vacant housing units were significantly associated with drug mortality changes among females, individuals aged 35–54, and Hispanic individuals. Changes in unemployment rates were significantly associated with drug mortality changes among the total population, females, and non-Hispanic White individuals. Changes in median household income were significantly associated with drug mortality changes in the total population and all sociodemographic subgroups. In addition, as shown in Table 2, Table 3 and Table 4, changes in the percentage of females were significantly associated with drug mortality changes among the total population and all sociodemographic subgroups, except individuals aged 55+ years and Hispanic individuals. We also observed in Table 2, Table 3 and Table 4 that changes in the percentage of people ≥ 65 years were positively associated with drug mortality changes, regardless of its significance, but the exception was among individuals aged 55+ years. An increase in the percentage of people ≥ 65 years was associated with decreased drug mortality rates among individuals aged 55+ years (see Table 3). Changes in the percentage of non-Hispanic Whites were consistently associated with drug mortality changes across Models 1–3 at alpha 0.05 among the total population and non-Hispanic White individuals, but not in other sociodemographic subgroups.

**Figure 2:**
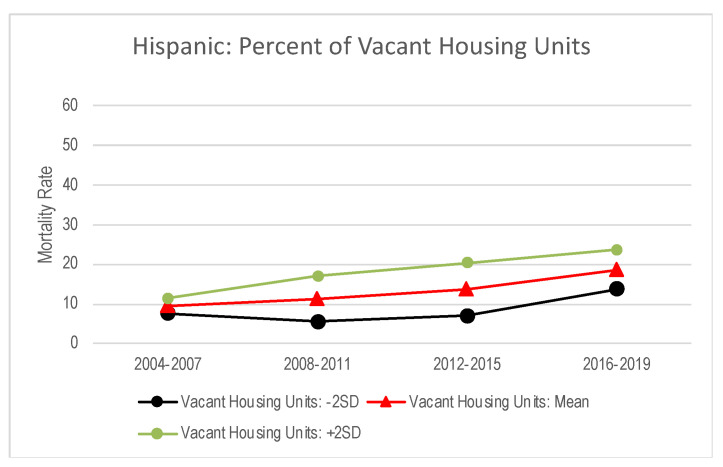
Predicted trajectories of county-level drug mortality rates with varying levels of percentage of county-level vacant housing units among Hispanic individuals: The Wide-Ranging Online Data for Epidemiologic Research, 2004–2019. Note: as shown in Table 2, Table 3 and Table 4, the trajectories of drug mortality rates were predicted, holding all other covariates within the model at their average value. Drug mortality rate indicates county-level crude drug mortality rates per 100,000 persons.

The interaction between time and the percent of vacant housing units was significant among Hispanic individuals (results not shown). To further examine the time interaction effects, we visualized drug mortality changes when the percent of vacant housing units was at 2 SD below the mean (i.e., low percentage of vacant housing units), mean (i.e., the average percentage of vacant housing units) and 2 SD above the mean (i.e., a high percentage of vacant housing units). As shown in Figure 2, while drug mortality rates were similar across different levels of the percentage of vacant housing units in 2004–2007 (i.e., prior to the recession), the extent of drug mortality changes differed by levels of the percentage of vacant housing units during and after the recession. Since the recession, counties with a high percentage of vacant housing units showed greater increases in drug mortality rates compared to counties with a low and average percentage of vacant housing units.

**Figure 3:**
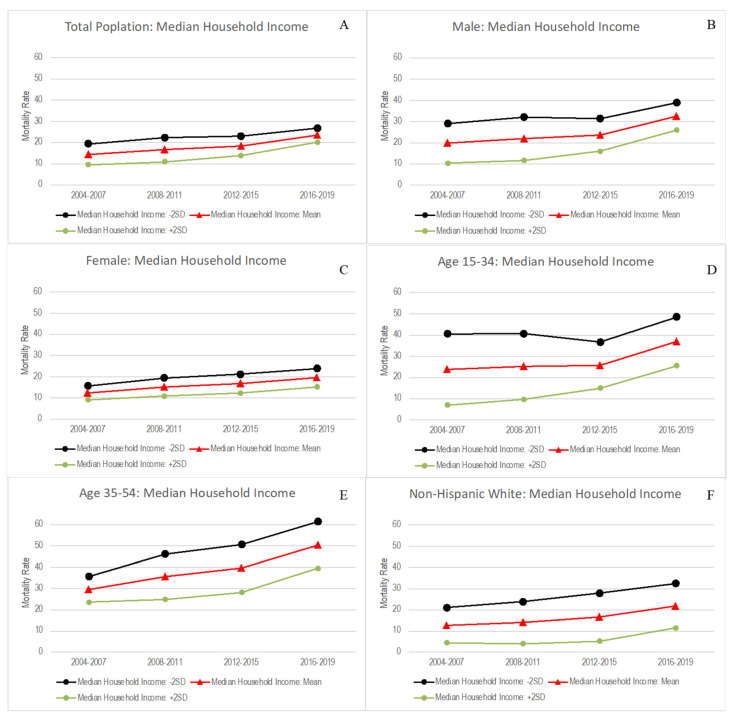
Predicted trajectories of county-level drug mortality rates with varying levels of county-level median household income among the total population and sociodemographic subgroups: The Wide-Ranging Online Data for Epidemiologic Research, 2004–2019. (**A**) Drug mortality rates by median household income levels in the total population; (**B**) Drug mortality rates for males by median household income levels; (**C**) Drug mortality rates for females by median household income levels; (**D**) Drug mortality rates for those aged 15–34 years by median household income levels; (**E**) Drug mortality rates for those aged 35–54 years by median household income levels; (**F**) Drug mortality rates for non-Hispanic White individuals by median household income levels.

In addition, we observed a significant interaction effect between time and median household income among the total population and some sociodemographic subgroups (results not shown). Figure 3 shows drug mortality changes from 2004–2007 through 2016–2019 when median household income was at 2SD below the mean, mean, and 2 SD above the mean in the total population (see Figure 3A), males (see Figure 3B), females (see Figure 3C), individuals aged 15–34 years (see Figure 3D), individuals aged 35–54 years (see Figure 3E), and non-Hispanic White individuals (see Figure 3F). The differences in drug mortality changes by the level of median household income became attenuated over time in the total population (Figure 3A), males (Figure 3B), and age 15–34 models (Figure 3D). On the other hand, in the female (Figure 3C), age 35–54 (Figure 3E), and non-Hispanic White models (Figure 3F), the differences in drug mortality changes by median household income levels increased following the recession.

## 4. Discussion

This study made one of the few attempts to examine the link between the changes in local economic conditions since the Great Recession and the recent county-level drug mortality trajectories over 16 years in the US. Our findings revealed that, among the total population, the crude death rates per 100,000 people increased from 13.9 deaths in 2004–2009 to 16 deaths in 2008–2011, 18 deaths in 2012–2015, 23 deaths in 2016–2019, indicating a 65% increase (see Figure 1). The increasing death rates since the early 2000s were found among the total population as well as all sociodemographic subgroups, consistent with recent studies [3,4,5].

We observed that changes in local economic conditions were associated with changes in drug mortality rates during and after the recession, as shown in Table 2, Table 3 and Table 4. Specifically, counties experiencing greater increases in median household income during and after the recession had a greater increase in drug mortality rates compared to counties with no or small increase in median household income among the total population and all sociodemographic characteristics (see Model 3 in Table 2, Table 3 and Table 4). While changes in median household income were significant in all models, changes in the percentage of vacant housing units and unemployment rates were significantly associated with drug mortality changes only in certain models (see Models 1 and 2 in Table 2, Table 3 and Table 4). The different associations across the county-level economic factors are due to different recovery patterns between labor and housing markets after the recession. The Great Recession fundamentally shifted many sectors of the US economy. This reorganization had significantly altered many local income distributions, with some outperforming their prerecession trend and others underperforming [40]. Conversely, most labor and housing markets have recovered since the recession, with less geographic variability than that of income distributions. Overall, our findings corroborate previous studies reporting significant associations between county-level economic conditions and drug misuse events and mortality [9,10,11,12,13,25,26,27] and the effects of unemployment rates during and after the recession on individual-level drug use [24].

In addition, our findings showed that associations of changes in the percentage of vacant housing units and unemployment rates with drug mortality changes differed by sociodemographic subgroups. The results from the fixed effects model in the total population were similar to those from the non-Hispanic White model (see Table 4) but different from those from other sociodemographic subgroup models (see Table 2, Table 3 and Table 4). For example, while changes in unemployment rates were significantly associated with drug mortality changes in the total population (see Model 2, Table 2) and non-Hispanic White models (see Model 2, Table 4), the changes were not significant among non-Hispanic Black and Hispanic individuals (see Model 2, Table 4). This indicates that results from the total population tend to reflect non-Hispanic White individuals’ experiences and are not generalizable to people of all sociodemographic characteristics.

Our results showed that females, individuals aged 35–54 years, and non-Hispanic White overdose death rates became more sensitive to county economic changes since the recession. Differences in drug mortality rates by levels of median household income became larger since the recession compared to before the recession among these subgroups (see Figure 3C,E,F). On the other hand, we found large differences in drug mortality rates by levels of median household income during the recession in the total population, males, and individuals aged 15–34 years, but the differences began to converge after the recession (see Figure 3A,B,D). We were not able to test what led to the difference in the association by sociodemographic characteristics, necessitating further investigation. As part of the potential reasons for the results among individuals aged 35–54 years, it may be because the working-age population is most sensitive to socioeconomic changes [24] and because the recent drug epidemic was more pronounced among this population [1,2]. Future research is suggested to examine factors contributing to age, gender, and racial/ethnic differences in the time interaction effect of changes in median household income.

Our study has limitations. Because this study is ecological in design (i.e., all variables were measured at the county level), the findings cannot be applied to the individual level. Also, we used the Decennial Census 2000 (before the recession), the ACS 2008–2012 (during the recession), and the ACS 2012–2016 and 2016–2020 (after the recession) to calculate county-level economic conditions prior to, during, and after the recession. This limited our ability to capture county-level economic conditions immediately prior to and right after the beginning of the recession.

## 5. Conclusions

Despite the limitations, this study extends current knowledge by examining the association between three measures of county-level economic changes following the 2008 recession and drug mortality over 16 years while controlling for time-variant county sociodemographic characteristics and removing all time-invariant differences across counties. Our findings offer important implications for the development of spatially targeted economic revitalization initiatives. As we continue to observe the lingering effects of the Great Recession and experience worldwide economic contractions due to the COVID-19 pandemic, there is a critical need to pay special attention to the county-level economic conditions to alleviate drug mortality and its geographical disparities. Public health efforts to reverse the drug epidemic will be more effective when coupled with a holistic approach to recognizing the perpetual effect of economic recession on local income distributions and creating economically viable and health-promoting communities.

## Figures and Tables

**Table 1:** Descriptive statistics.

	2000		2010 ^a^		2012–2016		2016–2020	
	Mean	95% CI	Mean	95% CI	Mean	95% CI	Mean	95% CI
Percent female	50.7	50.6–50.8	50.4	50.4–50.5	50.4	50.3–50.5	50.3	50.2–50.4
Percent of people ≥ 65 years	13.5	13.3–13.7	14.6	14.5–14.8	16.5	16.3–16.7	18.2	18.0–18.4
Percent of non-Hispanic Whites	81.9	81.0–82.7	78.4	77.6–79.3	83.6	82.9–84.3	81.7	81.0–82.4
Percent of college graduates	18.2	17.8–18.6	21.3	20.9–21.8	22.7	22.2–23.1	24.4	23.9–24.9
Percentage of vacant housing units	11.7	11.3–12.1	14.8	14.4–15.3	15.3	14.9–15.8	15.3	14.9–15.7
Median household income	37,868	37,393–38,343	47,606	46,995–48,219	49,688	49,054–50,323	57,345	56,620–58,070
Unemployment rates	5.8	5.7–6.0	9.4	9.3–9.5	7.6	7.4–7.7	5.4	5.4–5.5

^a^ County socioeconomic information for 2010 was obtained from the American Community Survey 2008–2012.

**Table 2:** Association between county economic changes and changes in county drug mortality rates from 2004–2007 to 2016–2019: Overall and sex-specific mortality rates.

	Overall Mortality (*n* = 1833)						Sex-Specific Mortality											
							Men (*n* = 1637)						Female (*n* = 1429)					
	Model 1		Model 2		Model 3		Model 1		Model 2		Model 3		Model 1		Model 2		Model 3	
	b	SE	b	SE	b	SE	b	SE	b	SE	b	SE	b	SE	b	SE	b	SE
Percent female	**−1.66**	0.42	**−1.70**	0.42	**−1.86**	0.42	**−3.87**	0.82	**−3.95**	0.81	**−4.31**	0.81	**−3.74**	0.56	**−4.00**	0.55	**−4.12**	0.55
Percent of people ≥ 65 years	0.70	0.39	0.63	0.39	0.42	0.39	1.12	0.65	1.02	0.66	0.64	0.66	**1.14**	0.43	**1.07**	0.43	**0.86**	0.43
Percent of non-Hispanic Whites	**−1.01**	0.35	**−0.93**	0.35	**−0.79**	0.35	**−1.43**	0.58	**−1.35**	0.59	−0.98	0.59	−0.43	0.35	−0.38	0.35	−0.29	0.35
Percent of college graduates	3.39	0.57	**3.42**	0.57	**4.03**	0.58	**6.38**	0.97	**6.29**	0.97	7.55	0.99	**2.70**	0.61	**2.49**	0.60	**3.07**	0.62
Percentage of vacant housing units	−0.03	0.49	-		-		0.81	0.85	-		-		**2.08**	0.55	-		-	
Unemployment rates	-		**0.56**	0.22	-		-		0.61	0.38	-		-		**0.68**	0.24	-	
Median household income	-				**−2.25**	0.43	-		-		**−4.07**	0.71	-		-		**−2.08**	0.44

Note. Bold indicates statistical significance *p*-value at alpha 0.05. SE indicates standard errors clustered by county.

**Table 3:** Association between county economic changes and changes in county drug mortality rates from 2004–2007 to 2016–2019: Age-specific mortality rates.

	Age 15–34 (*n* = 1198)						Age 35–54 (*n* = 1554)						Age 55+ (*n* = 957)					
	Model 1		Model 2		Model 3		Model 1		Model 2		Model 3		Model 1		Model 2		Model 3	
	b	SE	b	SE	b	SE	b	SE	b	SE	b	SE	b	SE	b	SE	b	SE
Percent female	**−3.24**	1.40	**−3.63**	1.39	**−4.47**	1.39	**−10.77**	1.35	**−11.3**	1.35	**−11.60**	1.35	−0.33	0.93	−0.38	0.93	-0.53	0.93
Percent of people ≥ 65 years	**2.19**	1.06	**2.12**	1.06	1.45	1.06	**5.08**	1.11	**4.88**	1.12	4.60	1.13	**−2.22**	0.67	**−2.31**	0.67	**−2.35**	0.67
Percent of non-Hispanic Whites	−1.49	0.78	−1.48	0.79	−0.97	0.78	−0.95	0.93	−0.86	0.93	**−0.54**	0.93	−0.81	0.43	−0.77	0.44	−0.73	0.44
Percent of college graduates	**10.01**	1.47	**9.70**	1.46	11.79	1.49	**8.63**	1.61	**7.92**	1.61	**9.28**	1.64	**3.60**	0.95	**3.55**	0.95	**3.98**	0.98
Percentage of vacant housing units	2.41	1.33	-		-		**6.14**	1.40	-		-		0.93	0.84	-		-	
Unemployment rates	-		0.44	0.61	-		-		1.09	0.63	-		-		0.59	0.40	-	
Median household income	-		-		**−6.32**	1.03	-		-		**−4.88**	1.15	-		-		**−1.34**	0.65

Note. Bold indicates statistical significance *p*-value at alpha 0.05. SE indicates standard errors clustered by county.

**Table 4:** Association between county economic changes and changes in county drug mortality rates from 2004–2007 to 2016–2019: Race/ethnicity-specific mortality rates.

	Black (*n* = 377)						White (*n* = 1788)						Hispanic (*n* = 289)					
	Model 1		Model 2		Model 3		Model 1		Model 2		Model 3		Model 1		Model 2		Model 3	
	b	SE	b	SE	b	SE	b	SE	b	SE	b	SE	b	SE	b	SE	b	SE
Percent female	**−6.33**	2.32	**−6.58**	2.32	**−6.96**	2.29	**−1.64**	0.49	**−1.69**	0.49	**−1.89**	0.49	−1.26	1.53	−1.34	1.54	−2.62	1.51
Percent of people ≥ 65 years	1.51	2.03	1.42	2.04	1.08	2.01	0.29	0.44	0.19	0.44	−0.08	0.44	1.66	1.10	2.13	1.08	1.72	1.05
Percent of non-Hispanic Whites	0.68	1.09	0.64	1.09	0.99	1.07	**−2.36**	0.45	**−2.23**	0.45	**−2.00**	0.45	**−0.99**	0.48	**−0.98**	0.49	−0.71	0.47
Percent of college graduates	**4.60**	2.15	**4.39**	2.12	**7.11**	2.23	**3.44**	0.65	**3.49**	0.65	**4.28**	0.66	1.71	1.93	1.65	1.94	**3.82**	1.92
Percentage of vacant housing units	1.68	2.26	-		-		−0.25	0.56	-		-		**2.79**	1.22	-		-	
Unemployment rates	-		0.46	1.04	-		-		**0.73**	0.25	-		-		-0.61	0.73	-	
Median household income	-		-		**−4.74**	1.30	-		-		**−2.91**	0.49	-		-		**−5.14**	0.96

Note. Bold indicates statistical significance *p*-value at alpha 0.05. SE indicates standard errors clustered by county.

## Data Availability

Publicly available datasets were analyzed in this study. These data can be found here: https://wonder.cdc.gov/.
